# Profiling Atlantic salmon B cell populations: CpG-mediated TLR-ligation enhances IgM secretion and modulates immune gene expression

**DOI:** 10.1038/s41598-018-21895-9

**Published:** 2018-02-23

**Authors:** Shiferaw Jenberie, Hanna L. Thim, J. Oriol Sunyer, Karsten Skjødt, Ingvill Jensen, Jorunn B. Jørgensen

**Affiliations:** 1 Norwegian College of Fishery Science, Faculty of Biosciences, Fisheries & Economics, University of Tromsø – The Arctic University of Norway, Tromsø, Norway; 2 Department of Pathology, School of Veterinary Medicine, University of Pennsylvania, Philadelphia, Pennsylvania 19104 USA; 3 Department of Immunology and Microbiology, Institute of Medical Biology, University of Southern Denmark, Odense, Denmark

## Abstract

While TLR-activated pathways are key regulators of B cell responses in mammals, their impact on teleost B cells are scarcely addressed. Here, the potential of Atlantic salmon B cells to respond to TLR ligands was shown by demonstrating a constitutive expression of nucleic-acid sensing TLRs in magnetic sorted IgM^+^ cells. Of the two receptors recognizing CpG in teleosts, *tlr9* was the dominating receptor with over ten-fold higher expression than *tlr21*. Upon CpG-stimulation, IgM secretion increased for head kidney (HK) and splenic IgM^+^ cells, while blood B cells were marginally affected. The results suggest that CpG directly affects salmon B cells to differentiate into antibody secreting cells (ASCs). IgM secretion was also detected in the non-treated controls, again with the highest levels in the HK derived population, signifying that persisting ASCs are present in this tissue. In all tissues, the IgM^+^ cells expressed high MHCII levels, suggesting antigen-presenting functions. Upon CpG-treatment the co-stimulatory molecules *cd83* and *cd40* were upregulated, while *cd86* was down-regulated under the same conditions. Finally, *ifna1* was upregulated upon CpG-stimulation in all tissues, while a restricted upregulation was evident for *ifnb*, proposing that salmon IgM^+^ B cells exhibit a type I IFN-response.

## Introduction

Adaptive immunity, present in all jawed vertebrates, is based on broad repertoires of antigen binding receptors expressed on B and T cells. In addition, B cells possess the capacity to directly sense and respond to pathogens through pattern recognition receptors (PRRs), including Toll-like receptors (TLRs). The development of different B cell subsets is determined in synergy between the B cell receptor, other receptor-derived signals and downstream signaling pathways^1^. These processes, as well as the various functionally distinct B cell subsets, are well studied in higher vertebrates, but less is known about the characteristics of teleosts B cells.

Teleosts and mammals diverged more than 350 million years ago; despite similarities between their immune systems, distinct differences are present. Lacking both bone marrow and lymph nodes, the anterior kidney (or head kidney; HK), and spleen are the organs involved in teleost B cell maturation and differentiation^2^. IgM, IgD and IgT are the only immunoglobulin (Ig) classes identified in fish^3,4^. IgM is regarded as a universal vertebrate Ig and was the first teleost Ig to be characterized^5^. Teleost IgM is a structural and functional homolog to mammalian IgM and the most prevalent isotype in fish serum present as a tetramer^6^. IgT (IgZ in cyprinids) is a unique teleost Ig^7–9^ involved in mucosal immunity analogous to mammalian IgA^10–13^. Compared to IgM and IgT, teleost IgD functions are less examined, however, in channel catfish (*Ictalurus punctatus*) IgD is suggested to be a mediator of innate immunity^14^. Two B cell populations were initially described in rainbow trout (*Oncorhynchus mykiss*); one subset expressing both IgM and IgD and one expressing only IgT^13^. These two B cell types are localized differently, with IgM^+^ cells being the main B cell population in spleen, kidney and blood^15^, while IgT^+^ cells dominate at mucosal sites^12,13,16^. Recently a third subset; expressing only IgD and homing mainly to the gills, was also identified in trout^17^.

Besides mediating humoral adaptive responses, mammalian B cells secrete cytokines and present antigen to T cells^18,19^. Naïve mammalian B cells are generally divided into three subsets; follicular B cells, B-1 B cells and marginal zone (MZ) B cells. Mature follicular B cells home to follicles in the secondary lymphoid organs (lymph nodes and spleen), where they present T cell dependent antigens (TD) to T cells. MZ B cells and B-1 B cells, respectively, reside in the marginal zone of the spleen or in body cavities and both have important roles in T cell independent antibody (Ab) responses. These cells occupy a niche between innate and adaptive immunity, secreting broadly cross-reactive IgM Abs^20,21^. Upon innate signaling, e.g. TLR-ligands, both MZ- and B1-B cells rapidly differentiate into Ab-secreting plasma cells, while follicular B cells only do this in the context of prior BCR activation^22^. Whether teleost B cells fall into several functionally distinct subpopulations is presently unknown. However, some of the features that characterize mammalian B-1 cells are described for teleost B cells; for example a high phagocytic activity^23,24^ and constitutive expression of PRRs^25^. Previous in *vivo* studies by our group showed that the combination of the TLR-ligand CpG and poly I:C, increased the neutralizing Ab response to a virus vaccine in Atlantic salmon (*Salmo salar*)^26,27^. This prompted us to use CpG as a model to investigate Atlantic salmon B cell biology, a topic of particular interest due to the high economic value of this species in the aquaculture industry and the urgent need for more efficient virus vaccines. A question of particular interest was whether the TLR-ligand CpG could directly stimulate B cells *in vitro* to secrete Abs and also to show enhanced antigen presentation functions stemming from increased expression of costimulatory molecules or MHC II molecules.

IgM^+^ B cells that were obtained by magnetic activated cell sorting (MACS) were found to constitutively express nucleic acid sensing TLRs, providing a foundation for TLR ligands to aid in shaping salmon B cell responses. Indeed, upon CpG stimulation, IgM secretion was increased in IgM^+^ cells; with the highest induction in HK compared to spleen and the lowest secretion in blood. In addition, gene expression analysis showed that the capacity of salmon IgM^+^ cells to trigger type I interferon (IFN-I) responses and present antigen appeared to be modulated by CpG stimulation. The results presented here provide a platform for further in-depth studies, dissecting different B cell subsets in teleost fish and their functional capacities related to humoral immunity, antigen presentation and regulatory functions.

## Results

### IgM^+^ B cells are the dominating B cell population in salmon kidney, blood and spleen

**Figure 1:**
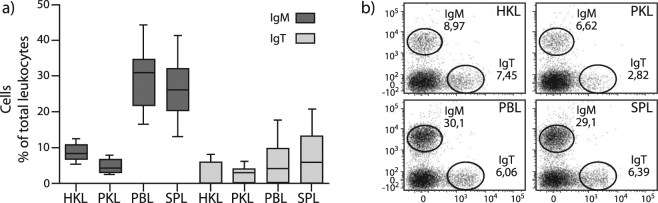
IgM^+^ cells are the dominating B cell population in Atlantic salmon systemic lymphoid tissues. Flow cytometry analysis of Atlantic salmon head kidney (HKL), posterior kidney (PKL), peripheral blood (PBL) and spleen (SPL) leukocytes stained with trout anti-IgM and IgT mAbs. (**a**) Median frequencies of IgM^+^ and IgT^+^ B cells of total leukocytes (n = 12). The box indicates 25^th^ and 75^th^ percentiles and the bars min and max values. (**b**) Representative flow cytometry dot plots showing the IgM and IgT percentages in the systemic lymphoid tissues.

The percentage of IgM^+^ and IgT^+^ B cells in relation to total leukocytes in salmon HK, posterior kidney (PK), peripheral blood (PB) and spleen were analyzed by flow cytometry using trout anti-IgM and anti-IgT mAbs (Fig. 1). For all tissues, the most abundant B cell population was the IgM^+^ B cells (Fig. 1a,b). The IgM^+^ population constituted about 30% of all leukocytes. In PB and spleen, and had a higher abundance compared to HK and PK (~5–10%). Both IgM^+^ and IgT^+^ cells showed a larger individual variation in PB (17 to 44% and 0.1 to 18%, respectively) and spleen (13 to 41% and 0.1 to 21%, respectively), that was not seen in the HK or PK. In four to five of the individuals analyzed, there were less than 2% IgT^+^ cells, which was evident in all tissues.

### Purity and viability of MACS sorted IgM^+^ B cells from HK, spleen and PB

**Figure 2:**
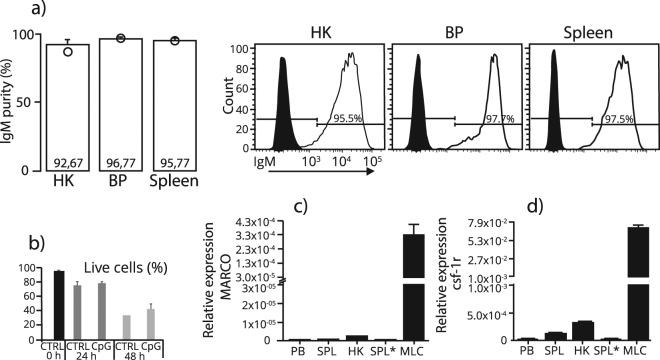
Purity and viability of IgM^+^ B cells sorted by magnetic activated cell sorting (MACS). (**a**) Upon sorting, the mean percentages of IgM^+^ cells from HK, PB and spleen (n = 3 for each tissue) were analysed by flow cytometry. The circle (○) represents total percentage of viable cells before gating for IgM^+^ events. Histogram represents one representative individual for each tissue, where IgM^+^ events are presented by the transparent peak and non-stained events by the black peak. (**b**) Viability of IgM^+^ cells kept in culture with or without CpG for 0, 12 and 24 hours. (**c** and **d**) The relative expression of MARCO and *csf-1r* in MACS and FACS sorted IgM^+^ cells, and in macrophage-like cells (MLC).

To study B cell biology of salmon, cultures of IgM^+^ cells were obtained by MACS. Before proceeding to further experiments, a basic characterization of these cells was done by purity and viability testing. As shown by flow cytometry, the purity of the IgM^+^ B cells was >95% for PB and SP and >92% for HK (Fig. 2a). Viability was 98% after MACS and decreased to 78 and 35% after 24 and 48 hours in culture, respectively. Viability in CpG stimulated IgM^+^ cells was in the same range as in unstimulated cells (Fig. 2b).

Since macrophages bind IgM through their Fc-receptor, there might be a possibility of macrophage contamination within the IgM^+^ MACS purified cells. To test this, the expression levels of genes encoding the scavenger receptor MARCO and the *csf-1r*, known as markers of monocyte-macrophage lineages in mammals^28^ and fish^29^, were analyzed in freshly MACS-purified HK, spleen and PB B cell populations. For comparison, the expression of the same genes was tested in HK-derived untreated macrophage-like cells (MLC), and as expected high mRNA levels of both genes were seen (Fig. 2c,d). Notably, while MARCO expression was undetected in IgM^+^ cells derived from PB and spleen (Cq cut-off to 37.8), and at very low levels in the HK cells (Cq > 36), a modest *csf-1r* expression was apparent in cells from all three tissues (Cq = 30–34), and again, HK IgM^+^ cells yielded the highest expression (Supplementary Fig. S1). A comparison of the relative expression of MARCO and *csf-1r* between the IgM^+^ cells and the MLC are presented in Fig. 2c,d. A 324, 122, and 282 fold higher expression of MARCO was found in the MLC compared to PB, HK and spleen, respectively (Fig. 2c). In the same tissues, the *csf-1r* was 2690, 217 and 560 fold higher expressed in the MLC than in the IgM^+^ cells, respectively (Fig. 2d). In FACS-sorted splenic IgM^+^ cells (n = 5), both MARCO and *csf-1r* were expressed about the same level as in MACS-sorted cell, Cq > 37.8 and >32, respectively (Supplementary Fig. S1). Additionally, the expression of the T cell marker *cd4–2*^30^ was explored and found to be undetectable across the tissues (Cq cut-off set to 38) (Supplementary Table S2). Our results indicate an absence of contaminating T cells in the sorted IgM^+^ populations, while traces of myeloid marker genes were detectable, most prominently in the HK derived cells.

### Atlantic salmon IgM^+^ B cells express high levels of *tlr9* and *tlr*8*a1*

**Figure 3:**
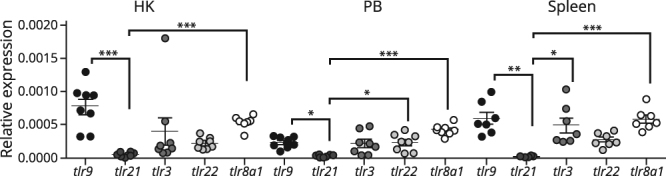
Relative expression of nucleic acid-sensing TLRs in Atlantic salmon IgM^+^ B cells derived from head kidney, peripheral blood (PB) and spleen. *tlr* expression was analyzed by RT-qPCR in IgM^+^ B cells sorted by MACS (n = 6 to 8). Data are presented as relative expression from individual fish (dots) where the bars indicate mean and s.e.m. Statistical significance between the *tlr* expression levels are indicated by brackets and the asterisks indicates the strength of significance: *p < 0.05 **p < 0.01 ***p < 0.001.

Characterization of the basal TLR expression in salmon B cells from lymphoid organs is key to understanding how TLR signaling may affect salmon B cell responses. Here, we focused on a set of nucleic acid recognizing TLRs shown to respond to ss-RNA (TLR8a1), ds-ssRNA (TLR3 and 22)^31^ and CpG DNA (TLR9 and 21)^32^. *tlr3*, *8* and 9 are orthologues to mammalian TLRs, whereas *tlr21* and 22 are absent in mammals. Figure 3 shows that all five *tlr*s were expressed by the IgM^+^ B cell populations and that *tlr8a1* and *tlr9* had the highest relative expression, representative across all tissues. *tlr*21 was the lowest expressed *tlr*-gene, and was respectively 18, 9 and 23-fold less expressed compared to *tlr9* in HK (p < 0.001), PB (p < 0.05) and spleen (p < 0.01) IgM^+^ B cells. The individual variation of *tlr8a1*, one of several teleost *tlr8* isotypes^33^, was low both within and across tissues. For the two *tlr*s recognizing dsRNA, the expression of the fish specific *tlr22*, was relatively stable across tissues and individuals, while the *tlr3* expression displayed a higher individual variation.

### CpG-stimulation alters *sigm* transcript levels in IgM^+^ B cells

Constitutive expression levels of both *tlr9* and 21 transcripts were evident when analyzing the MACS- sorted IgM^+^ B cells; hence, this was followed up by investigating if their ligand CpG would affect the expression pattern of selected immune genes. For all the assayed genes, the effect of non-CpG treatment was negligible or minimal (Supplementary Fig. S2). The basal expression level of secreted IgM (*sigm*) was higher across the three tissues compared to membrane bound IgM (*migm*) (Supplementary Table 2). Higher basal levels of *sigm* transcripts (about 16-fold) were observed in IgM^+^ HK (mean Cq = 18.2, SD = 0.3) and spleen (mean Cq = 18.3, SD = 0.1) compared to PB (Mean Cq = 22.5, SD = 0.2) B cells.

**Figure 4:**
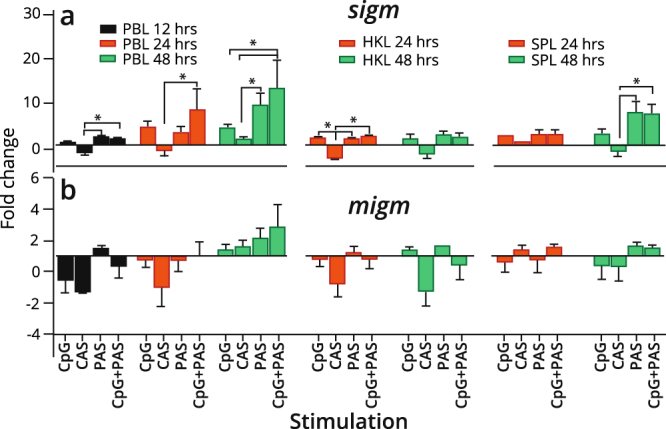
Expression of B cell markers genes in IgM^+^ B cells treated with CpG and/or condition supernatants. IgM^+^ B cells from peripheral blood (PB), head kidney (HK) and spleen leukocytes were MACS sorted and analyzed by RT-qPCR for expression of (**a**) secreted IgM (*sigm*) and (**b**) membrane IgM (*migm*). Gene expression data at each time point were normalized against the reference gene EF1aB and fold changes were calculated using the unstimulated sample at the same time point^74^. Data represent mean ± s.e.m. from at least three individuals (n = 3 to 6). The line intersecting the y-axis at 1 represents the unstimulated control that the fold change of the treatments are in relation to. Significant fold changes (p < 0.05) are indicated by *. CAS; Control adherent cell supernatant, PAS; Pulse adherent cell supernatant.

In general, the control supernatant, CAS, provided the weakest transcript induction, or even inhibited the expression of the B cell marker genes compared to the untreated control. Similarly, neither CpG nor PAS exhibited significant induction of *migm* in any of the tissues over time (Fig. 4b). For *sigm*, a gradual increase in induction was observed over time in B cells derived from PB and spleen (Fig. 4a). The CpG alone and the combination treatment, respectively, displayed fold inductions of 3.8 and 12.8 for PB, and 3.4 and 7.6 for spleen at 48 hours. Significant increase in *sigm* mRNA levels compared to the CAS treatment (p < 0.05) were observed in PB B cells stimulated with PAS or the combination at 12 hours, which was still significant in the combination treatment, at 24 and 48 hours (p < 0.05). The *sigm* transcript regulation was least affected by the stimulations in HK derived IgM^+^ B cells, although a significant (p < 0.05) increase was apparent in all the three stimulations compared to CAS at 24 hours. Of notice, while HK response was unaffected over time, for spleen B cells the PAS and combination stimulation yielded a significant induction of *sigm* (p < 0.05) compared to CAS at 48 hours.

### Induction of professional antigen presenting cell marker genes in IgM^+^ B cells by CpG

**Figure 5:**
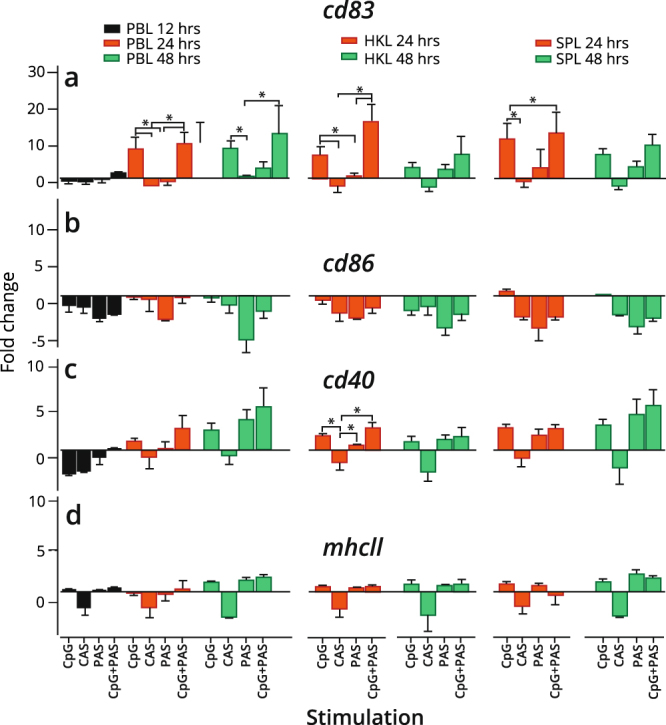
Expression of APC marker and co-stimulatory genes in IgM^+^ B cells treated with CpG and/or condition supernatants. IgM^+^ B cells from Atlantic salmon peripheral blood (PB), head kidney (HK) and spleen leukocytes were MACS purified, stimulated *in vitro* or left untreated and analyzed by RT-qPCR for expression of (**a**) *cd83*, (**b**) *cd86*, (**c**) *cd40* and (**d**) *mhcII*. Gene expression data at each time point were normalized against the endogenous control gene EF1aB and fold changes were calculated using the unstimulated sample at the same time point^74^. Data represent mean ± s.e.m from at least three individuals (n = 3 to 6). The line intersecting the y-axis at 1 represents the unstimulated control that the fold change of the treatments are in relation to. Significant fold changes (p < 0.05) are indicated by *. CAS; Control adherent cell supernatant, PAS; Pulse adherent cell supernatant.

The capabilities of B cells to phagocytose and present antigens (Ag) have been described in trout^34^ and zebrafish^19^. Hence, we hypothesized that IgM^+^ B cells from Atlantic salmon may express distinct transcripts of selected Ag presentation and co-stimulatory genes upon CpG stimulation. Accordingly, we measured the relative expression of *cd83, cd86, cd40* and *mhcII* in IgM^+^ B cells obtained from the three tissues. CpG alone and the combination treatments augmented the expression of *cd83*, a marker for mature dendritic cells (DCs) and activated B cells^35^. For the three tissues, significant increase in *cd83* expression was evident in CpG and combination compared with CAS and PAS at 24 hours (Fig. 5a). However, in HK and spleen, *cd83* transcript levels slightly declined after 24 hours for the CpG and combination treatments (Fig. 5a), while the expression was still significantly maintained after 48 hours in PB IgM^+^ B cells. *cd86* is one of the best-defined co-stimulatory molecules in mammals and plays a major role in providing co-stimulation to T cells by both DCs and B cells^36^. Recently, the involvement of CD86 in B cell-initiated adaptive immunity in a zebrafish model was reported^19^. Interestingly, except for CpG treated spleen B cells, the expression of *cd86* was downregulated compared to the control for all the treatment groups and time points in HK and PB (Fig. 5b). CD40 is a transmembrane glycoprotein and a member of the TNF receptor superfamily expressed by APCs^37^. Interactions between CD40 and its ligand, CD40L, holds a major role in the cross-talk between T cells and APCs. CpG and PAS, both as standalone stimulations, and in combination, induced a modest, although comparable expression of *cd40* transcripts in sorted IgM^+^ B cells from the three tissues (Fig. 5c). Significantly (p < 0.05) higher induction of *cd40* was observed in CpG and PAS stimulated HK B cells at 24 hours compared to CAS. *mhcII* was constitutively expressed in IgM^+^ B cells from all the assayed tissues (basal level expression of Cq = 20.7, SD = 0.4 for PB; Cq = 20.9, SD = 0.8 for HK; Cq = 20.9, SD = 0.9 for spleen). At transcript level (Fig. 5d), the stimulations did not markedly affect the regulation of *mhcII* although the CAS simulation resulted in a more pronounced, yet insignificant, downregulation.

### CpG-stimulation induce antibody secretion in HK and spleen IgM^+^ B cells

**Figure 6:**
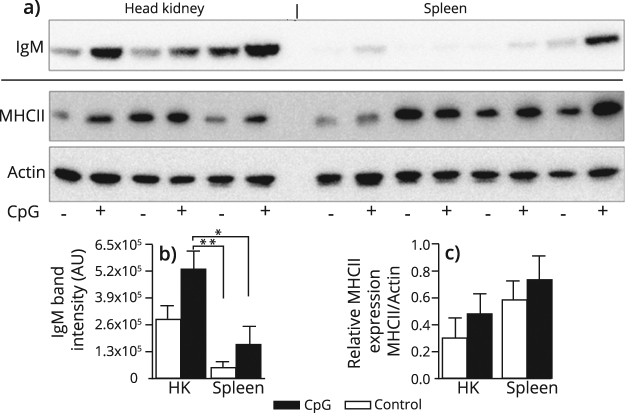
Protein level analysis of IgM and MHCII expression by Western blot. MACS purified IgM^+^ B cells from head kidney (HK) and spleen were treated with CpG (+; 2 µM) or left untreated (−) for 72 hours before harvest of supernatants and cell lysates. Figures present results from two independent experiments. (**a**) Representative Western blot analysis showing IgM secretion in supernatants (upper panel), and MHCII expression in cell lysates (middle panel). An anti-actin Ab was applied as loading control (lower panel). MHCII and actin were run on the same gel; after MHCII detection, the gel was washed and reprobed with the anti-actin Ab. Bar graphs showing the mean band intensity (AU) of (**b**) secreted IgM and (**c**) MHCII relative expression in HK and spleen B cells (n = 6). Significantly higher IgM secretion is indicated by * where *p < 0.05 and **p < 0.01. The original full blot images can be found in Supplemental Fig. S5.

To validate the qPCR results, the IgM content in cell culture supernatants and the MHCII expression in cell lysates from MACS-purified B cells were analyzed by Western blot. In line with the transcript data, translational results revealed that IgM secretion was enhanced in CpG treated cells compared to untreated controls (Fig. 6a,b). A pronounced individual variation in IgM secretion was observed for both controls and stimulated cells. Of notice, significantly higher levels of secreted IgM were detected in CpG-stimulated HK B cells when compared to the spleen cells (Fig. 6b). The lowest levels of secreted IgM were detected in PB B cell culture supernatants (Supplementary Fig. S4). At protein level, CpG-treatment affected MHCII expression modestly, although not significantly different, compared with the control cells (Fig. 6c).

### CpG-stimulation induces type I *ifn* gene expression in IgM^+^ B cells

**Figure 7:**
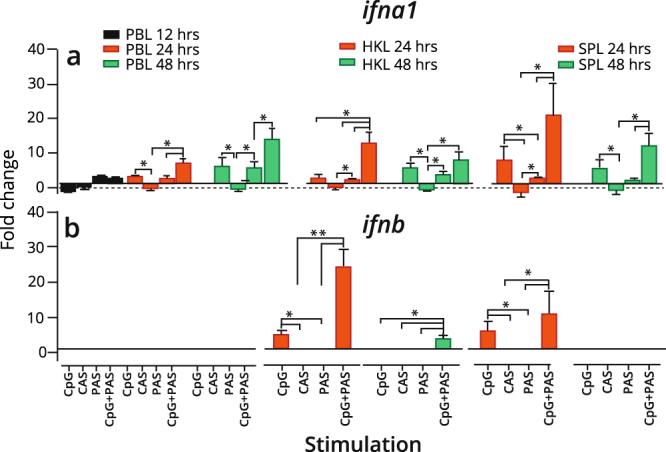
Expression of type I IFN genes in IgM^+^ B cells treated with CpG and/or condition supernatants. IgM^+^ B cells from Atlantic salmon peripheral blood (PB), head kidney (HK) and spleen (SP) leukocytes were MACS sorted, stimulated *in vitro* or left untreated and analyzed by RT-qPCR for expression of (**a**) *ifna1* and (**b**) *ifnb* genes. Gene expression data at each time point were normalized against the endogenous control EF1aB and fold changes were calculated using unstimulated cells at the same time point^74^. Data represent mean ± s.e.m from at least three individuals (n = 3 to 6). The line intersecting the y-axis at 1 represents the unstimulated control that the fold change of the treatments are in relation to. Significant fold changes, p < 0.05 or p < 0.01, are indicated by * or **, respectively. CAS; Control adherent cell supernatant, PAS; Pulse adherent cell supernatant.

To assess the capability of salmon IgM^+^ B cells to induce type I IFN responses, the expression of type I *ifn* genes were measured. CpG alone significantly (p < 0.05) up-regulated *ifna1* transcripts in B cells derived from the systemic tissues. The combination of CpG and PAS, however, induced a more pronounced expression than the standalone stimulation, which suggests an additive effect (Fig. 7a). Over time, *ifna1* expression patterns were similar for HK and spleen B cells and declined at 48 hours post stimulation, while the expression in PB IgM^+^ B cells was kept significantly higher at the same time point.

Upregulation of *ifnb* transcripts was observed only in IgM^+^ B cells obtained from HK and spleen at 24 hours for the two CpG treatments and was highly significant (p < 0.01) compared to CAS, while the expression was undetectable in PB IgM^+^ B cells (Fig. 7b). The basal expression of *ifnb* was undetected in IgM^+^ B cells obtained from all the tissues (Cq cut-off set to 36), while the expression of *ifnc* (Supplementary Table S2) was undetectable both in controls and stimulated IgM^+^ B cells.

## Discussion

A better understanding of basic B cell biology, such as responsiveness to potential vaccine adjuvants and the mechanisms mediating their activity, is crucial for the development of new vaccines for farmed fish. For Atlantic salmon, many aspects of B cell functionality are unknown, partly due to the lack of population specific mAbs. Here we have used mAbs available against trout IgM and IgT combined with flow cytometry to separate and quantify B cell subpopulations in systemic immunological organs of non-immunized salmon. The monoclonal IgF1–18 (6-1-18) anti-trout IgM Ab is earlier reported to recognize both salmon IgM isotypes^38^, while the cross-reactivity of the anti-trout IgT mAb to salmon IgT is reported herein (Supplementary Fig. S3)

Single IgM and IgT positive cells were identified in total leukocytes from PB, spleen, HK and PK at percentages varying from three to 44% for IgM and between 0.1 to 21% for IgT. The data are in accordance with a similar study in trout^13^ demonstrating markedly higher abundances of IgM^+^ B cells compared to IgT^+^ B cells in systemic lymphoid tissues. As reported previously in several other teleost fish species^13,39,40^ and also in salmon^38^, PB and spleen here harbored the highest number of IgM^+^ cells, ~30% of total leukocytes. The percentages of IgT^+^ cells were in general low, and across the tissues investigated, several individuals had less than two percent of IgT^+^ B cells. The trout anti-IgT mAb is directed against the constant heavy chain domains two to four of IgT subclass 1^13^ and was recently shown to cross-react with the other two trout IgT subclasses^41^. Three IgT subclasses are present in salmon as well, showing _~_80% identity both to each other and to trout IgT^9^. Western blot analysis of a Flag-tagged CH4-domain of salmon IgT overexpressed in HEK293 cells verified cross-reactivity of the trout IgT Ab to salmon IgT (Supplementary Fig. S3). Whether the trout Ab is able to recognize all three salmon IgT^+^ B cell subclasses is presently unknown, thus we cannot conclude if one or all three salmon IgT subclasses are present in the investigated tissues.

Induction of protective Ab responses is a major goal for the development of prophylactic vaccines for farmed fish. Studies by us and other groups have reported the potential of CpG-based adjuvants for fish vaccines^26,27,42^. A combination of the TLR-ligands CpG and poly I:C formulated in a whole-virus vaccine for salmon induced high titer-protective Abs after vaccination^26,27^. However, how these TLR ligands potentiate Ab responses in bony fish remain poorly understood. One important scope of this study was to investigate the direct effects of TLR engagement on salmon B cell functions. In this context, we used purified IgM^+^ B cells obtained by MACS purification. While these cells were devoid of T cell marker genes, traces of well-known monocyte-macrophage lineage marker genes were detected. However, since their numbers were very low, their contribution to the overall mRNA levels expressed in the cultivated cells is most likely modest. Of notice, macrophage marker-gene expression were comparable between MACS and FACS purified IgM^+^ cells, suggesting that the levels of B cell purity by the two methods are comparable. Since bony fish B cells are suggested to have a myeloid origin^34^, one could speculate on whether myeloid restricted proteins like CSF-1R are totally absent in B cells.

To date, the expression pattern of TLRs in fish has mainly been monitored in whole tissues^43–45^, while knowledge regarding their expression in defined leukocyte subsets are scarce. Here, transcripts of the five nucleic-acid binding TLRs were detected in the purified IgM^+^ B cells from the three tissues. This suggests that salmon B cells have the potential to respond directly to CpG DNA and ds/ssRNA. The relative expression levels for each TLR showed little variability across the different tissues; slightly elevated levels of *tlr9* and t*lr8a1* were detected compared to the other TLRs, while *tlr21* was the least expressed TLR. A similar study in trout reported higher constitutive levels of *tlr9* and *tlr8a2* compared to *tlr3* in IgM^+^ positive B cell populations from blood, kidney and spleen^25^, although such differences were less apparent in our data. Variations in fish species, age and cell sorting procedures may explain the dissimilarities between these results. As in salmon, the fish specific receptor, *tlr22*, was abundant in trout B cells from all three tissues^25^. The most profound difference reported here is between *tlr9* and *21*, where *tlr9* had 10 to 30-fold higher basal expression levels compared to *tlr21*. TLR21 is a non-mammalian TLR, first identified in chicken, with functions resembling TLR9^46,47^. Later, *tlr21* genes have been identified in several teleost species including salmonids^48,49^. In zebrafish, both TLR9 and 21 respond to CpG-ODNs and slight differences in their ligand recognition profiles are shown with TLR9 possessing a broader sequence specific range compared to TLR21^32^. Whether this holds true for other fish species, including salmonids, warrants further investigations.

An important question for this study was whether CpG stimulation could activate salmon B cells to become Ab secreting cells (ASCs). Secreted IgM protein was detected in non-stimulated IgM^+^ cells from spleen and HK, with markedly higher levels in HK derived B cells compared to spleen (Fig. 5a,b). In contrast, IgM secretion from PB IgM^+^ cells were hardly detected (Supplementary Fig. S4). RT-qPCR correspondingly demonstrated that non-treated IgM^+^ cells from PB expressed the lowest levels of secreted *igm* transcripts (mean Cq of 22.5), while basic levels in HK and spleen derived cells were about 16-fold higher (mean Cq of 18.2 and 18.3, respectively). The presence of Ig-secreting cells in the absence of immune activation suggest that plasmablasts/plasma cells might be present in these organs, and probably at a much higher abundance in HK compared to spleen and blood. This observation is in agreement with previous reports for other teleosts^50,51^ and suggest that ASCs may persist from previous antigenic exposures or that these cells spontaneously secrete IgM, thus resembling the mammalian B-1 cells. In our study, the levels of secreted IgM protein increased upon *in vitro* CpG stimulation, especially for HK derived cells. The levels of s*igm* transcripts in the IgM^+^ cells from the three tissues interrelated with the levels of secreted IgM in cell supernatants, where only secreted Ig transcripts derived from HK cells were significantly higher than the controls. The lack of salmon plasma cell markers impeded further characterization of the ASCs. For B cells activated through the BCR prior to cell harvest, CpG may contribute to terminal differentiation towards plasma cells. Whether CpG alone exerts an effect on naïve B cells, or, alternatively, that the combination of BCR triggering by the anti-IgM Ab and TLR signaling elicits differentiation into ASCs, is still an open question.

In addition to promote Ab responses, TLR signaling is involved in B cell cytokine secretion and antigen presentation^52^. In our data, the *mhcII* gene-expression pattern in IgM^+^ cells was similar across all the three tissues, and its levels were non-significantly upregulated (~2-fold) by the stimulations (Fig. 4d). The detection of MHCII protein by Western blotting in lysates from IgM^+^ cells validated the finding (Fig. 5b). In general, information about the role of teleost B cells as APCs is lacking. It has been reported that a population of IgM^+^/MHC-II^+^ cells from salmon HK has the ability to accumulate ovalbumin antigen *in vitro*^53^, thus, supporting a role of HK-derived B cells in the antigen-presenting process. In this study, *cd83* and *cd40* transcript levels were slightly upregulated in CpG-treated HK-derived IgM^+^ cells (Fig. 5a,c), while at the same time *cd86* levels decreased (Fig. 5b). These results were observed for all tissues. However, in contrast to the earlier study^53^, where the B cells were cultivated and stimulated within a heterogeneous leukocyte population and subsequently FACS-sorted, the purified B cells were here directly stimulated. Our current data show that CpG alone, or possibly in combination with BCR stimulation, is sufficient to affect the gene expression pattern of central antigen presenting markers on salmon B cells. The rapid up-regulation of *cd83* transcripts in the B cells described herein is also observed in murine B cells after TLR-engagement or BCR ligation^54^. While *cd83* mRNA expression was solely upregulated in CpG-treated B cells, both CpG alone and PAS induced elevated *cd40* mRNA levels in all the tissues when compared to the CAS, although significantly different only for HK at 24 hours. This is similar to the situation in mammals where both TLR signaling and cytokines derived from myeloid cells regulate *cd40* expression in B cells^55^. Results based on micro-array data, have shown that a suite of genes encoding secreted proteins, which includes cytokines, chemokines and TNF receptor superfamily members, are expressed in salmon adherent leukocytes stimulated with the same CpG ODN as used here^56^. It is therefore likely that PAS contains molecules with a potential to selectively upregulate different B cell transcripts.

The high phagocytic capacity of teleost B cells^24,57^, combined with the upregulation of different APC markers reported here and by others^19,53,58^, suggest that TLR-mediated activated B cells are capable of modulating T cell responses. As in our study, high expression of surface MHCII levels and *cd83* transcripts for LPS-stimulated trout splenic B cells has been reported elsewhere^58^, underscoring their role as APCs. However, the down-regulation of *cd86* in our study contradicts the upregulation of this gene obtained in the previous study^58^. This may suggest that B cells show both similar and contrasting responses to the two microbial molecules CpG and LPS, which may influence their functions. Another important aspect is that the LPS-stimulation in the previous work^58^ was not performed on purified B cells, and hence, the activation by other cell types and their secretory products may directly or indirectly influence B cell responses. The enhanced effect of the PAS supernatant when used with CpG, as reported herein, underscores that molecules derived from other cell types significantly shape the B cell responses to TLR ligation.

Trout peritoneal IgM^+^ cells, upon *in vivo* exposure to *E. coli* or VHSV, are shown to differentiate towards IgM-secreting cells, and at the same time these cells show decreased levels of MHCII surface expression compared to non-treated controls^59^. As the terminal differentiation of mammalian plasma cells is shown to be accompanied by the loss of MHCII expression^29^, these authors argue that decreased MHCII surface levels may be a coincident indicator for trout plasma cell differentiation. This somewhat differs from our data where CpG treatment of IgM^+^ cells did not significantly affect MHCII levels, while increased IgM secretion clearly indicated CpG-induced differentiation towards plasma cells. However, the specific stage of differentiation of these cells towards the terminal plasma cell was not identified. Most of these cells may be at the plasmablast stage and might not have accomplished MHCII downregulation after 72 hours *in vitro* CpG treatment.

In our data, high individual variations in IgM secretion and MHCII proteins levels were apparent for both controls and stimulated IgM^+^ cells. This heterogeneity in responses suggest variations in the B cell composition between the individual donor fish. As a result, the secreted IgM may reflect both the presence of previously activated plasmablast/plasma cells and naïve B cells responding to the TLR-ligation. The impact of CpG treatment on MHCII levels may be different on naïve versus previously activated B cells. This proposed heterogeneity in the B cell populations restricts the usage of our data to evaluate if any association between plasma cell development and MHCII levels exists. Anyhow, whether active suppression of class II genes is an underlying mechanism for bony fish plasma cell differentiation or not, is an interesting question that calls for future investigation only attainable using subpopulation specific mAbs.

In general, information on CD40 signaling in teleost B cells is limited. However, studies in both zebrafish^60^ and salmon^61^ have reported that structural characteristics of CD40 and its ligand (CD40L or CD154) are conserved between mammals and fish. Salmon *cd40* was upregulated in cultivated HK leucocytes after exposure to different PAMPs including CpG^61^, which is in accordance with the present data. In zebrafish, CD40/IgM double positive lymphocytes were reported^60^, suggesting that CD40 is present in teleost B cells. The present work further support this, showing that salmon HK, spleen and PB B cells express considerable levels of *cd40* (Supplementary Table S2). Opposite to *cd40* and *cd83*, decreased levels of *cd86* were apparent upon CpG treatment across the tissues, which may suggest a mechanism to avoid prolonged T cell activation^62^.

Teleost fish possess a well-developed type I IFN system encoded by multiple IFN genes. Six IFN-I subtypes are identified in the salmon genome, where the subtypes *ifna*, *ifnb* and *ifnc* are the best-defined representatives^63^. Here, CpG-stimulated B cells derived from HK and spleen showed increased transcript levels of *ifnb*, while elevated *ifna1* mRNA levels were detected in B cells from all three tissues (Fig. 7). Together, these results propose that salmon B cells exhibit a type I IFN-response. In line with this, rainbow trout IgM^+^ PB and spleen cells have been shown to transcribe *ifn1* upon poly I:C and VHSV infection^64^ and type I IFN responses have been reported for CpG B treated murine B cells^65^. In general, the basal levels of IFN transcripts in the purified salmon B cells were low (*ifna1* mean Cq 31.8–33.7) or non-detectable for *ifnb* and i*fnc* (Supplementary Table 2). Interestingly, elevated *ifna1* mRNA levels were detected both for CpG alone and for CpG + PAS during the course of time, while *ifnb* levels peaked at 24 hours and then declined to its basal levels at 48 hours. A possible explanation for this is that the initial *ifna1* burst triggers the transcription of interferon regulatory factors, which mediate a positive feedback loop that lead to the induction of a second wave of *ifna1* transcription^66,67^. The cytosolic receptors RIG-I/MDA5 that recognize viral RNA are suggested to be the main receptors to activate pathways leading to *ifna1* expression in salmon^68^. Our results imply that other receptors specific for DNA induce *ifna1* gene transcription in salmon B cells. The inverted (GpC) ODN control was non-stimulatory (Supplementary Fig. S2), proposing that the *ifna1* induction in salmon IgM^+^ cells is mediated through a CpG-specific TLR. Unlike *ifna1*, *ifnb* is reported to show modest basal expression in most salmon organs, while a rapid and potent induction appear in salmon HK and spleen upon treatment with R848, a ligand for TLR7/TLR8^68^. This observation, together with the rapid increase in *ifnb* levels in the purified HK and spleen B cells upon TLR9 ligation, suggest that signaling through the TLR7 family activates pathways important for *ifnb* expression in salmon leukocytes. The significant increase in *ifnb* expression when combining PAS and CpG, suggest that other signals provided by the supernatant are necessary for optimal *ifnb* production in B cells.

B cells have important protective roles against many viral infections, mainly through the production of neutralizing Abs. In mammals type I IFN promotes B cell activation and Ab responses, including class switch during viral infections^69,70^. It is thus possible that type I IFNs produced by salmon B cells, affect the regulation and functional activities of the B cells themselves, in addition to that of other immune cells. Interestingly, by using a DNA vaccine model, adjuvant effects of Atlantic salmon type I IFNs were demonstrated and shown to increase the virus specific IgM response^71^. These results establish a link between type I IFNs and the adaptive immune system in salmon, although the mechanisms behind are currently unknown. In support, our group has earlier reported that CpG and poly I:C significantly enhance virus-specific humoral responses *in vivo*, where a substantial increase in *ifna1* transcripts in HK and spleen were detected at 12 and 48 hours post injection^26^. In the previous study^26^, however, whether the IFNs was produced by B cells or by other cell types was not investigated. The implications of direct interactions between type I IFNs and B cells in salmon has not been elucidated and is a topic for future studies.

## Materials and Methods

### Fish

Atlantic salmon (*Salmo salar* L.) roe from Aqua Gen (Aqua Gen, Kyrksæterøra, Norway) was hatched and smoltified at Tromsø Aquaculture Research Station (Tromsø, Norway). The fish were kept in seawater at natural temperature and photoperiod and fed with dry commercial feed (Skretting, Stavanger, Norway) before transfer to the research facility of the Arctic University of Norway, Tromsø, where they were kept at similar conditions until sacrifice. Fish were acclimated for at least two weeks before sampling. Two batches of fish were used; 400 g fish for FACS analysis and 1000–1500 g fish for MACS sorting and immune gene expression analysis of IgM^+^ B cells. We confirm that the experimental protocols used for the live fish experiments were based on the Animal Welfare Act (https://www.regjeringen.no/en/dokumenter/animal-welfare-act/id571188/) and performed in accordance with relevant guidelines and regulations given by the Norwegian Animal Research Authority.

### Reagents

Phosphorothioate-modified B class CpG 2006 ODN (5′-T*CG T*CG T*TT T*GT C*GT T*TT G*TC G*T*T-3′, * indicates phosphothioate bonds) and non-CpG 2007 ODN, inverting all CGs to GCs, were purchased from Integrated DNA technologies. The monoclonal Abs IgF1-18 (6-1-18) anti-trout IgM^38^ and anti-trout IgT^13^ are described earlier. Isotype specific secondary Abs; IgG1-RPE (for IgM) and IgG2a-APC (for IgT) were both from Jackson ImmunoResearch. Fixable Viability Dye 780 (FVD780) was purchased from eBioscience. MACS MS columns and anti-Mouse IgG1 MicroBeads were purchased from Miltenyi Biotec. Anti-actin Ab was purchased from Sigma, while the preparation of MHCIIβ antiserum is described previously^56^.

### Cell isolation

Leukocytes from HK, PK, spleen and PB were isolated on Percoll (GE Healthcare) gradients^72,73^. Briefly, HK and spleen were sampled aseptically and kept on ice-cold transport medium (L-15 medium with 10 U/ml penicillin, 10 μg/ml streptomycin, 2% fetal bovine serum (FBS), 20 U/ml heparin) until homogenization on 100-μm cells strainers (Falcon). The resulting cell suspensions were layered on 25/54% discontinuous Percoll gradients and centrifuged at 400 × *g* for 40 min at 4 °C. Cells at the interface were collected and washed twice in L-15 medium. PB was collected from the caudal vein using heparinized vacutainer tube and diluted immediately at least two fold in ice-cold transport medium. The resulting suspension was layered on a 54% Percoll gradient, centrifuged, and harvested as above. Cells were counted using an automatic cell counter (NucleoCounter, YC-100). The isolation of HK monocytes/macrophages were performed as described elsewhere^56^ and the adherent cells were cultivated in L-15^+^ (L-15 supplemented with 5% FBS and penicillin/streptomycin) in 24 well plates (Nunclon Delta Surface, Thermo Scientific) for 3 days before being analyzed.

### Flow cytometry analyses

For the Ig-frequency analysis, 3 × 10^5^ HK, PK, PB and spleen leukocytes from twelve individuals were each pelleted at 500 × *g* directly after isolation, further washed in PBS^+^ (PBS with 0.5% BSA) and stained with anti-trout IgM (1:200 dilution) and anti-trout IgT (2 μg/mL) mAbs for 30 minutes. After washing with PBS^+^, the samples were incubated with isotype specific secondary Abs; IgG1-RPE and IgG2a-APC (1:400 dilution), respectively, and FVD780 (1 μl/ml) in PBS^+^ for 20 minutes. All staining and centrifugation steps were done at 4 °C in 96-well U-bottom plates (Nunclon Delta Surface). Ig-frequencies were analyzed on Aria II flow cytometer (Becton Dickinson), while data processing was done in FlowJo (Tree Star). Dead cells (FVD780^+^) and doublets (SSC-A vs SSC-H) were excluded before the frequency of IgM and IgT were determined

### Magnetic activated cell sorting (MACS) of IgM^+^ B cells

The sorting procedure was performed as per the company’s recommendation (Miltenyi Biotec). All washes and incubations were done using MACS sorting buffer (SB; PBS with 0.5% bovine serum albumin and 2 mM EDTA) and spun at 450 × *g* 4 °C for 5 minutes. Briefly, after one wash with SB, 7–8 × 10^7^ cells were incubated with a 1:200 dilution of anti-trout IgM mAb for 30 minutes at 4 °C. After two washes, cells were incubated in 80 μl SB and 20 μl anti-mouse IgG1 microbeads for 15 minutes at 4 °C. Sorted IgM^+^ B cells were pelleted immediately and resuspended in L-15^+^. Zero time-point samples were obtained before initiation of stimulations. A time course study of viability of cultured IgM^+^ MACS purified cells from blood was performed by flow cytometry using the FVD780 stain as described above (n = 2). The cells were seeded and stimulated with CpG (2 μM) as described below. Purity of sorted IgM^+^ B cells was determined by flow cytometry and RT-qPCR of *cd4–2* (T cell marker), macrophage colony stimulating factor receptor (*csf-1r*) and the scavenger receptor MARCO genes transcripts. For comparison, the expression of the same genes was measured in HK-derived untreated MLC and FACS sorted IgM^+^ splenic cells, where MLC were excluded by gating.

### Stimulation of sorted IgM^+^ B cells for gene expression study

MACS sorted IgM^+^ B cells, 1 × 10^6^, were seeded in 100 μl L-15^+^ in 96-well U-bottom plates (Nunclon Delta Surface, Thermo Scientific). For stimulation, either PAS or CAS (see below) in a 1:2 dilution or 2 μM CpG B ODN was applied to the cells. In addition, some cells were stimulated with PAS (diluted 1:2) combined with CpG (2 μM). Controls received only L-15^+^. A non-CpG control (2 µM) was also included. Cells were incubated at 14 °C for 12, 24 or 48 hours before harvested for RNA isolation. Due to variations in cell yield at each isolation, the number of individuals for each treatment varied between 3 to 6.

### Preparation of conditioned supernatant

Six million HKLs were pulsed with 2 μM CpG 2006 for 8 hours (pulse adherent cell supernatant; PAS) or left unstimulated (control adherent cell supernatant; CAS) at 14 °C. After the pulse treatment, culture media and non-adherent cells were removed, while the adherent cells were washed and further incubated at 14 °C for 72 hours. After centrifugation at 450 × *g* at 4 °C for five minutes, PAS and CAS, respectively, were harvested, pooled (three individuals) and stored at −80 °C until used.

### RNA extraction and transcript analyses

Total RNA from sorted IgM^+^ B cells and HK MLC was isolated using the RNeasy Mini Kit (Qiagen) following the manufacturer’s recommendation. On-column DNase digestion was performed using RNase-free DNase set (Qiagen), and RNA was quantified using NanoDrop (ND 1000 Spectrophotometer). Twenty-five microliter cDNA reactions with 150 ng total RNA were synthesized using TaqMan reverse transcription reagents (Applied Biosystems) under the following conditions: 25 °C for 10 min, 48 °C for 30 min and 95 °C for 5 min. cDNA samples were diluted 1:5 and stored at −20 °C until use.

qPCR was run as 20 μl duplicate reactions with 6 ng cDNA/reaction on a 7500 Fast Real-Time PCR Systems (Applied Biosystems) according to their standard protocol. Primer and probe sequences are listed in Supplementary Table 1. A negative control (no template) reaction was performed for each primer pair. The Cq-threshold was set to 0.20 for both reference gene EF1aB and target genes. A melt curve analysis was also performed to ensure that a single product had been amplified. For the stimulated cells, fold change was calculated using the non-treated cells from each tissue and time point as a control^74^. Relative expression (zero hour samples) of *tlr3*, 22, 9, 21 and 8a1 were calculated using the 2^−ΔCq^ method^75^ where ΔCq was calculated by subtracting the EF1aB Cq value from the target gene Cq value.

### Western blot analysis of MHC II and secreted IgM

Supernatants and cell lysates from non-treated or CpG (2 µM) treated HK (n = 6) and spleen (n = 6) sorted IgM^+^ B cells were harvested after 72 hours culture at 14 °C. Supernatants were up-concentrated by 0.5 ml centrifugal filter columns (Millipore) and then diluted 32-fold before denaturation in 2× LDS buffer at 70 °C for 10 minutes. Cells were sampled in 2× LDS buffer and diluted to 1.62 × 10^5^ cells/sample before denaturation. All samples were run on precasted 4–12% gradient NuPAGE Novex Bis-Tris gels and subjected to SDS-polyacrylamide gel electrophoresis (SDS-PAGE) with 1× MOPS buffer (Invitrogen) for 50 min at 200 V and 120 mA. MagicMark™ XP and SeeBlue Plus 2 pre-stained (both from Invitrogen), were loaded for molecular weight estimation. The proteins were blotted onto a polyvinylidene difluoride membrane, blocked with 5% BSA (Sigma) and incubated overnight with either anti-trout IgM Ab (1:200 dilution), anti-salmon MHCII Ab (1:1000)^56^ or anti-actin rabbit Ab (1:600). The respective blots were incubated for 1 hour with goat anti-mouse-HRP Ab or goat anti-rabbit-HRP Ab (1:8000 dilution; Santa Cruz Biotechnology) in 5% BSA. The blots were developed using SuperSignal West Femto Trial Kit (Thermo) and a KODAK Image Station 4000 MM Digital Imaging System. For IgM, band intensities (arbitrary units, AU) were determined by subtracting the background noise from the visualized bands, while the relative expression of MHCII was calculated by:

$$MHCII(Relative\,expression)=\frac{MHCII(AU\,IgM)-Noise(AU)}{Actine\,(AU)-Noise\,(AU)}$$

### Statistical analysis

RT-qPCR data were based on duplicate measurements of three to six individuals and were analyzed in GraphPad Prism 5.04 or SPSS version 24. Statistical evaluation were performed using two-tailed non-parametric Mann-Whitney U or Dunn’s multiple comparison test following a significant Kruskal-Wallis test on the fold change and relative expression of the transcript data, respectively. The IgM secretion and MHCII protein expression data were analyzed by Mann-Whitney U. For all analyses, differences were considered significant at p < 0.05.

## Electronic supplementary material

Supplementary Information 
