# Author Correction: Profiling Atlantic salmon B cell populations: CpG-mediated TLR-ligation enhances IgM secretion and modulates immune gene expression

**DOI:** 10.1038/s41598-018-24843-9

**Published:** 2018-04-19

**Authors:** Shiferaw Jenberie, Hanna L. Thim, J. Oriol Sunyer, Karsten Skjødt, Ingvill Jensen, Jorunn B. Jørgensen

**Affiliations:** 1 Norwegian College of Fishery Science, Faculty of Biosciences, Fisheries & Economics, University of Tromsø – The Arctic University of Norway, Tromsø, Norway; 2 Department of Pathology, School of Veterinary Medicine, University of Pennsylvania, Philadelphia, Pennsylvania 19104 USA; 3 Department of Immunology and Microbiology, Institute of Medical Biology, University of Southern Denmark, Odense, Denmark

Correction to: *Scientific Reports* 10.1038/s41598-018-21895-9, published online 23 February 2018

This Article contains errors in the Results section under subheading ‘IgM+ B cells are the dominating B cell population in salmon kidney, blood and spleen’.

“The IgM+ population constituted about 30% of all leukocytes. In PB and spleen, and had a higher abundance compared to HK and PK (~5–10%).”

should read:

“The IgM+ population constituted about 30% of all leukocytes in PB and spleen, and had a higher abundance compared to HK and PK (~5–10%).”

