# Supplementary material for: Profiling Atlantic salmon B cell populations: CpG-mediated TLR-ligation enhances IgM secretion and modulates immune gene expression
Source: Sci Rep. 2018 Feb 23;8:3565. doi: 10.1038/s41598-018-21895-9 (PMC5824956; doi:10.1038/s41598-018-21895-9)
Supplement: Supplementary file 1 — Supplementary Information [file 41598_2018_21895_MOESM1_ESM.doc]

**Supplementary information**

**Profiling Atlantic salmon B cell populations: CpG-mediated TLR-ligation enhances IgM secretion and modulates immune gene expression**

*Shiferaw Jenberie^1^, Hanna L Thim^1^, Oriol Sunyer^2^, Karsten Skjødt^3^, Ingvill Jensen^1^, Jorunn B Jørgensen^1^*

*^1^*Norwegian College of Fishery Science, Faculty of Biosciences, Fisheries & Economics

University of Tromsø – The Arctic University of Norway

*^2^*Department of Pathology, School of Veterinary Medicine, University of Pennsylvania, Philadelphia, Pennsylvania 19104, USA

*^3^*Department of Immunology and Microbiology, Institute of Medical Biology, University of Southern Denmark, Odense, Denmark

**Supplementary Figure S1.**


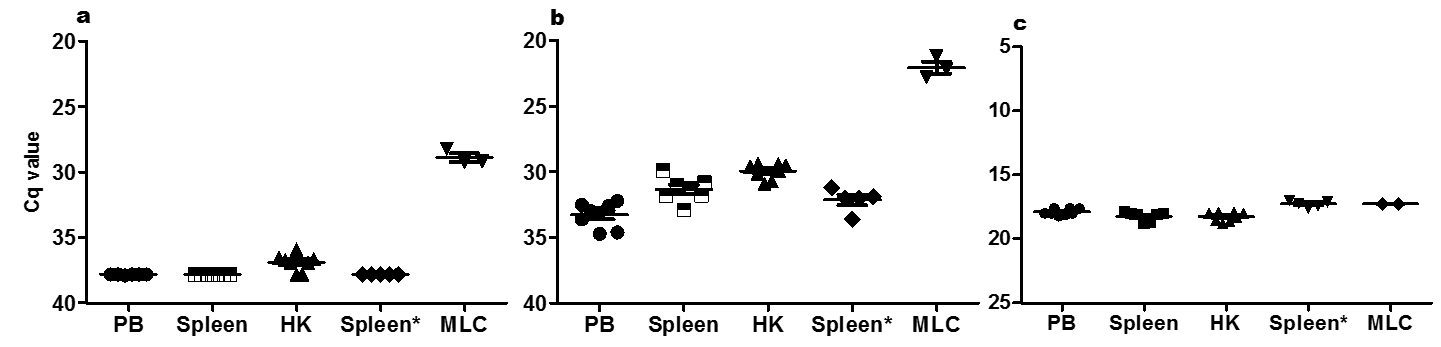


**Expression of putative macrophage marker genes in sorted IgM^+^ cells and MLCs of Atlantic salmon.** IgM^+^ cells were sorted either by MACS (n=8) from PB, spleen and HK or by FACS (Spleen*) (n=5) while MLCs (n=3) were obtained from HK as described in the materials and methods. Total RNA was extracted from unstimulated samples and equal amount of RNA was reverse transcribed and analyzed by qPCR for expression of a) macrophage scavenger receptor MARCO, b) colony stimulating factor-1 receptor (*csf-1r*) and c) the reference gene EF1aB. Scatter plots show individual values obtained from duplicate qPCR runs. Minus RT and melt-curve analysis were also performed to validate the result as described in the material and method section.

**Supplementary Figure S2.**


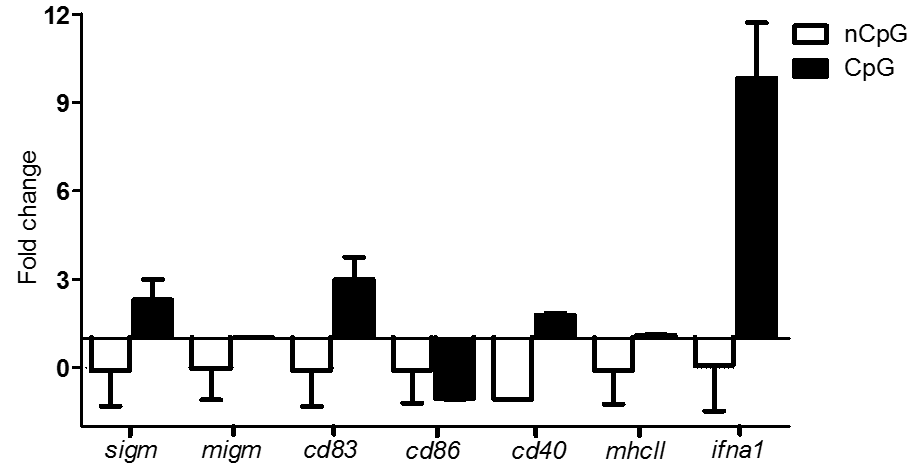


**Gene expression profile of MACS sorted IgM^+^ B cells treated with CpG or non-CpG (nCpG).** PB IgM^+^ B cells were sorted by MACS and cultured for 24 hours with CpG (2µM), non-CpG (2µM) or left untreated. Cells were harvested for RNA extraction and analyzed by RT-qPCR as described in the materials and methods. Gene expression data were normalized against a reference gene, EF1aB. Fold changes for *sigm, migm, cd83, cd85, cd40, mhcll and ifna1* were calculated as described in the materials and methods^1^. Data present mean ± s.e.m. from two independent experiments.

**Supplementary Figure S3.**


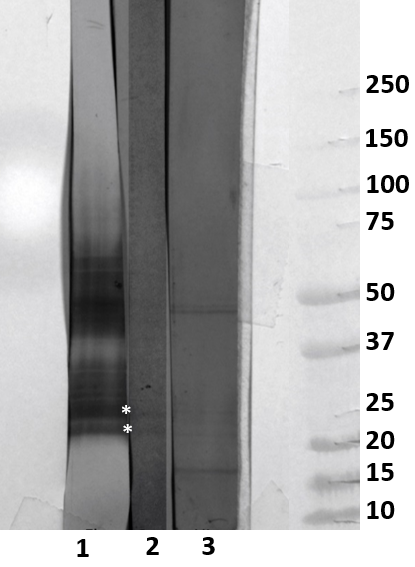


**Demonstrating the cross-reactivity of the anti-trout IgT mAb to the salmon IgT CH4 domain.** HEK293 cells were transiently transfected with an expression vector encoding the salmon IgT CH4 containing a Flag epitope tag (theoretical MW 21.2). The cells were lysed, the samples run on the same gel, blotted to a membrane that was cut into pieces (1,2,3) and developed by the three different Abs. **1)** α-Flag mAb (Rockland 200-301-B13, 0.125 µg/mL), **2)** α -trout IgT mAb (1:500**,** 2.6 µg/mL) and **3)**α-His mAb (MicroMol 295, 1:500, 2 µg/mL). The α-Flag mAb recognized two bands made up of the salmon IgT CH4-domain (lane 1, indicated by *). MS-analysis identified these bands as the salmon IgT CH4-domain, with and without signal peptide (Dr. Søren Grove, personal communication). Similar bands were weakly detected by the α -trout IgT mAb (lane 2), suggesting cross-reactivity of the anti-trout IgT mAb to the salmon IgT CH4 domain. The α-His mAb showed no binding to the two bands (lane 3). A range of proteins, probably representing the salmon IgT CH4-domain in various glycosylation ‘stages’, also appear in lane 1.

**Supplementary Figure S4.**

**Supplementary Figure S4.**

**
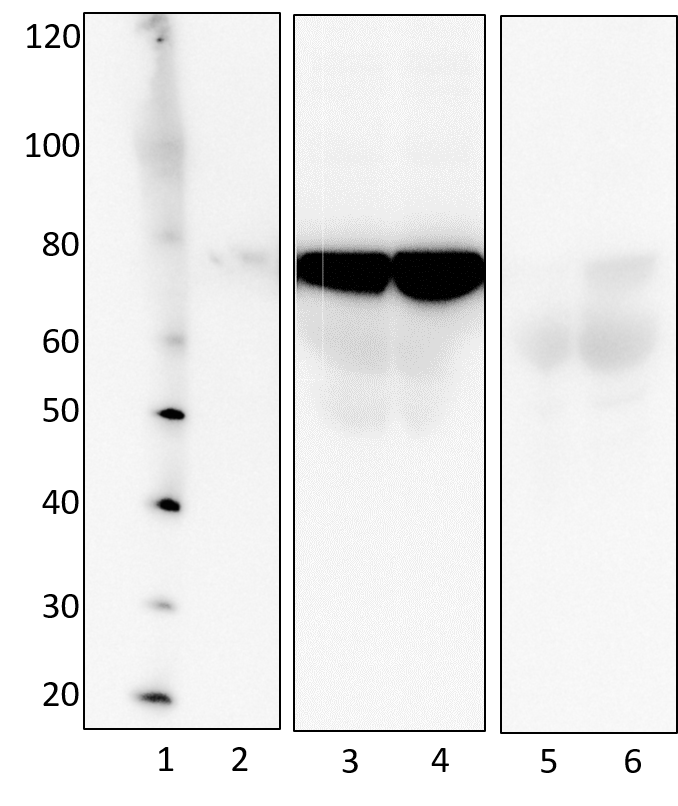
**

**CpG stimulation differentially modulates secretion of IgM from sorted IgM^+^ B cells**. MACS purified 6x10^6^ IgM^+^ cells from PB and spleen were seeded in 500 µl of L-15^+^ and stimulated with CpG (2µM) or left untreated. After 72 hours of incubation at 14 ^O^C, cells were span for 5 minutes at 400*xg* and culture supernatants were harvested. Supernatants were up-concentrated by 0.5 ml centrifugal filter columns (Millipore) and 2 µl of the up-concentrated material were run on precasted 4–12% gradient NuPAGE Novex Bis-Tris gels with molecular weight markers and subjected to SDS-PAGE as described in Materials and Methods. Proteins were blotted onto a polyvinylidene difluoride membrane, blocked with 5% BSA (Sigma) and incubated overnight with anti-trout IgM mAb (1:200 dilution). Blot was incubated for 1 hour with goat anti-mouse-HRP Ab (1:8000 dilution; Santa Cruz Biotechnology) in 5% BSA and developed using SuperSignal West Femto Trial Kit (Thermo) for 10 seconds. Lane 1 is a weight marker; lane 2 is affinity-purified salmon IgM from serum (positive control); lane 3 and 4 are unstimulated and stimulated IgM^+^ cells, respectively, from spleen; lane 5 and 6 are unstimulated and stimulated IgM^+^ cells, respectively, from PB. The samples were run on the same gel, however the gel has been cropped to remove lanes with non-relevant proteins as indicated by the white space between the wells.

**Supplementary Figure S5. Full blot images for Figure 6.**

IgM-exposed for 3 minutes


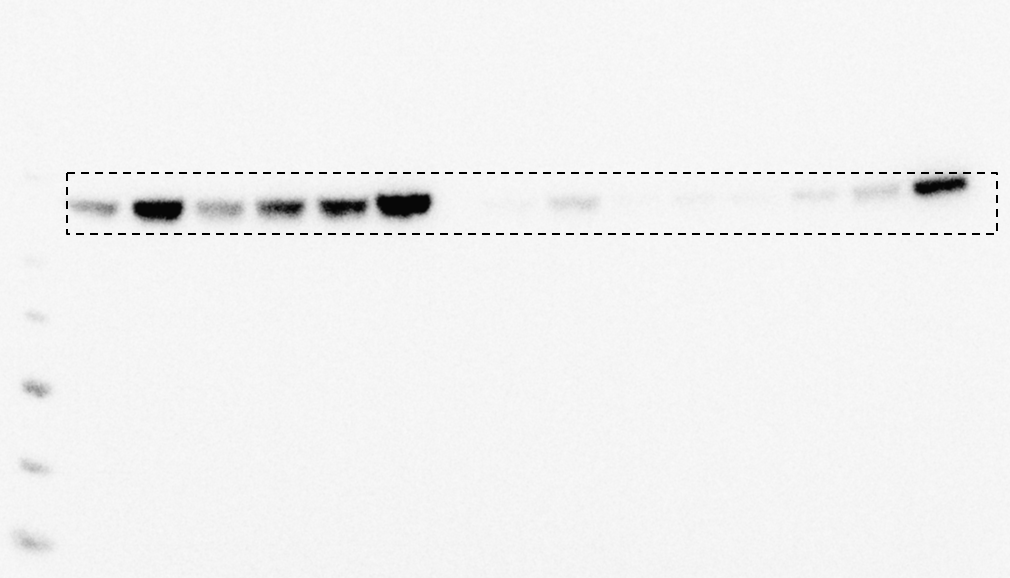


MHCII-exposed for 2 minutes

Actin-exposed for 30 seconds


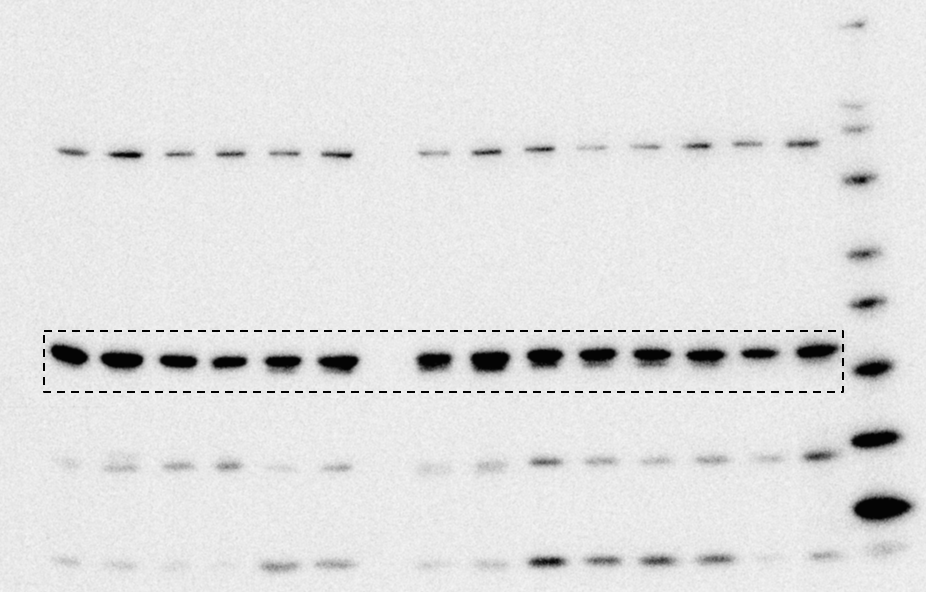


**Supplementary Table S1. Primers and probes used for RT-qPCR.**

| **Genes** | **Oligo name** | **Sequence (5'–3')** | **PCR efficiency** | **Accession No** |
| --- | --- | --- | --- | --- |
| *ef1aβ* | Fw | TGCCCCTCCAGGATGTCTAC | 1.9 | BG933897 |
|  | Rev | CACGGCCCACAGGTACTG |  |  |
|  | Probe | AAATCGGCGGTATTGG |  |  |
| MARCO | Fw | AGGACCTGCTGGTGTTAATG | 2.16 | XM_014173984.1 |
|  | Rev | CTGCTCTTTCACCCTTCTCTC |  |  |
| *Csf-r1* | Fw | CACCAGTAACCCTAACCACTTC | 2.00 | [NM_001171807.1](https://www.ncbi.nlm.nih.gov/entrez/viewer.fcgi?db=nucleotide&id=284520969) |
|  | Rev | GACCTGCTTGTCCTGCATTA |  |  |
| *cd4-2a* | Fw | TGCAAAGAAGGCGCAGAT | 1.76 | EU409793 |
|  | Rev | GAAAACCTTTAATTTAACAGG |  |  |
| *sigm* | Fw | CTACAAGAGGGAGACCGGAG | 2.04 | BT060420 |
|  | Rev | AGGGTCACCGTATTATCACTAGTTT |  |  |
|  | Probe | TCCACAGCGTCCATCTGTCTTTC |  |  |
| *migm* | Fw | CCTACAAGAGGGAGACCGA | 1.73 | S48658 |
|  | Rev | GATGAAGGTGAAGGCTGTTTT |  |  |
|  | Probe | TGACTGACTGTCCATGCAGCAACACC |  |  |
| *Tlr3* | Fw | TGGCTGAACGGAACAAACG | 1.87 | BK008646 |
|  | Rev | GGCGTGTTGCACACATACTCA |  |  |
|  | Probe | AGTGTGCCGGGCAT |  |  |
| *tlr8a1* | Fw | ACCAAAACCACTAATGACATCATCTTCA | - | FJ467615.1 |
|  | Rev | TGGTGATGCCATCAGGTATGTTT |  |  |
|  | Probe | CTCAGTCGACGCTCCTC |  |  |
| *tlr9* | Fw | TCTATGGCTGGGATGTCTGGTA | 2.03 | EF672331 |
|  | Rev | CAGTTGTGAGTAGCCCTTGTGT |  |  |
|  | Probe | CAGCACCTGGAAGCAG |  |  |
| *tlr21* | Fw | TCCTGGTTCGGCCAACA | 2.19 | HG514151 |
|  | Rev | TTGGCAGTGCTCCTCTTTCTC |  |  |
|  | Probe | TGGCGCAGGCTGA |  |  |
| *tlr22a* | Fw | GAGGGTCAAGGCTCTCACTGA | - | AM233509 |
|  | Rev | GCAGTCGGAGC-AGCTAAGTGT |  |  |
|  | Probe | ATTGCCTGCCATATAC |  |  |
| *cd83* | Fw | GTGGCGGCATTGCTGATATT | 2.01 | BT047309.2 |
|  | Rev | CTTGTGGATACTTCTTACTCCTTTGCA |  |  |
|  | Probe | CACCATCAGCTATGTCATCC |  |  |
| *cd86* | Fw | ACTTCACACTCGATTACGGCTGCT | 1.93 | XM_014215239.1 |
|  | Rev | AGCAGGAATAAGGTGACACACCGA |  |  |
| *cd40* | Fw | ATGCCATGCCAAGAGGGTGAAT | 1.91 | NM_001141236.1 |
|  | Rev | ATTTGCATGGGCTGAGGCTTGT |  |  |
| *mhcII* | Fw | AGAAGCCTGGAACAAAGGTCCTGA | 1.98 | EF451156.1 |
|  | Rev | AACTGTCTTGTCCAGTATGGCGCT |  |  |
| *ifna1* | Fw | CCTTTCCCTGCTGGACCA | 199 | XM_014187640.1 |
|  | Rev | TGTCTGTAAAGGGATGTTGGGAAAA |  |  |
|  | Probe | CTTTGTGATATCTCCTCCCATC |  |  |
| *ifnb* | Fw | TGCATTGGAGGCTATGCGATAT | 2.06 | EU735552.1 |
|  | Rev | TTCCCAAACACCACCTACGACA |  |  |
| *ifnc* | Fw | ATGTATGATGGGCAGTGTGG | - | NM_001279097.1 |
|  | Rev | CCAGGCGCAGTAACTGAAAT |  |  |

**Supplementary Table S2.** Mean and standard deviation (SD) of Cq values for the assayed genes. Samples were analyzed in duplicates with internal controls to control for inter-plate variations. Mean Cq values with SD for the zero hour time point (basal level expression), controls and stimulation groups are presented. PB-peripheral blood; ND; not detectable (Cq cut-off set to 36 for *ifnb*).

| **Genes** | **Tissues** | **Basal**  **expression** | **Control (Mean, SD)** | | | **CpG (Mean, SD)** | | | **CAS (Mean, SD)** | | | **PAS (Mean, SD)** | | | **CpG+PAS (Mean, SD)** | | |
| --- | --- | --- | --- | --- | --- | --- | --- | --- | --- | --- | --- | --- | --- | --- | --- | --- | --- |
|  |  |  |  |  |  |  |  |  |  |  |  |  |  |  |  |  |  |
|  |  |  | **12 hrs** | **24 hrs** | **48 hrs** | **12 hrs** | **24 hrs** | **48 hrs** | **12 hrs** | **24 hrs** | **48 hrs** | **12 hrs** | **24 hrs** | **48 hrs** | **12 hrs** | **24 hrs** | **48 hrs** |
|  |  | 18.7 | 19.2 | 20.3 | 21.3 | 18.9 | 19.8 | 19.8 | 19.2 | 20.7 | 22.0 | 18.6 | 20.2 | 20.7 | 18.6 | 19.6 | 19.4 |
| ***ef1aβ*** | **PB** | (0.4) | (0.2) | (0.9 | (0.8) | (0.1) | (0.7) | (0.3) | (0.2) | (1.0) | (1.4) | (0.0) | (1.3) | (0.6) | (0.1) | (1.0) | (0.1) |
|  |  | 19.2  (0.6) |  | 20.2  (0.3) | 21.3  (0.9) |  | 19.4  (0.3) | 19.9  (0.8) |  | 20.3  (0.3) | 20.9  (0.5) |  | 19.5  (0.3) | 20.5  (0.7) |  | 19.3 | 19.7 |
|  | **HK** |  |  |  |  |  |  |  |  |  |  |  |  |  |  | (0.3) | (1.0) |
|  |  | 19.2 |  | 19.9 | 21.2 |  | 19.2 | 19.7 |  |  | 21.7 |  | 19.0 | 20.8 |  | 18.8 | 19.6 |
|  | **Spleen** | (0.5) |  | (0.5) | (1.2) |  | (0.3) | (0.7) |  | 19.4 | (1.0) |  | (0.4) | (0.1) |  | (0.3) | (0.7) |
|  |  | 22.5 | 26.9 | 26.5 | 29.0 | 26.2 | 24.0 | 25.6 | 27.0 | 26.5 | 29.2 | 25.0 | 24.6 | 25.4 | 25.2 | 23.4 | 24.1 |
| ***sigm*** | **PB** | (0.2) | (0.0) | (1.7) | (1.5) | (0.4) | (0.9) | (1.6) | (0.1) | (0.9) | (0.6) | (0.4) | (1.5) | (1.4) | (0.2) | (0.4) | (1.2) |
|  | **HK** | 18.2  (0.3) |  | 22.3  (0.9) | 22.6  (1.7) |  | 20.4  (0.5) | 20.4  (0.8) |  | 23.3  (0.7) | 22.6  (0.7) |  | 20.7  (0.7) | 20.3  (0.9) |  | 20.7  (0.5) | 20.0  (0.7) |
|  |  | 18.3 |  | 23.1 | 25.8 |  | 20.9 | 22.9 |  | 23.9 | 26.3 |  | 20.5 | 22.7 |  | 20.4 | 21.7 |
|  | **Spleen** | (0.1) |  | (0.9) | (2.5) |  | (1.1) | (1.8) |  |  | (1.1) |  | (1.0) | (0.8) |  | (1.2) | (1.1) |
| ***migm*** |  | 26.6 | 29.4 | 28.8 | 30.7 | 29.5 | 27.9 | 28.8 | 29.7 | 28.2 | 32.2 | 28.1 | 27.3 | 29.3 | 28.7 | 29.5 | 27.5 |
|  | **PB** | (0.3) | (0.1) | (0.9) | (2.3) | (0.4) | (0.3) | (1.6) | (0.2) | (1.3) | (1.3) | (0.4) | (0.8) | (1.4) | (0.3) | (1.4) | (0.8) |
|  | **HK** | 27.0  (0.3) |  | 29.8  (1.1) | 30.9  (1.2) |  | 28.6  (0.9) | 28.8  (0.5) |  | 30.9  (0.9) | 31.2  (0.7) |  | 28.3  (0.8) | 29.1  (0.7) |  | 28.4 | 28.5 |
|  |  |  |  |  |  |  |  |  |  |  |  |  |  |  |  | (1.1) | (0.7) |
|  |  | 26.7 |  | 29.7 | 32.1 |  | 28.5 | 30.3 |  |  | 32.7 |  | 28.1 | 30.8 |  | 27.7 | 29.4 |
|  | **Spleen** | (0.1) |  | (0.8) | (2.1) |  | (0.9) | (1.7) |  | 30.7 | (1.5) |  | (0.7) | (0.5) |  | (0.8) | (1.0) |
| ***cd83*** | **PB** | 26.2  (0.8) | 30.0  (0.7) | 31.0  (1.7) | 34.0  (1.6) | 29.7  (0.6) | 28.0  (1.3) | 29.6  (2.1) | 30.0  (0.5) | 30.4  (0.5) | 35.3  (0.5) | 29.0  (0.6) | 30.0  (1.5) | 31.8  (1.7) | 28.3  (0.3) | 27.0  (1.2) | 29.3  (1.7) |
|  | **HK** | 26.2  (0.8) |  | 30.4  (1.0) | 31.8  (2.3) |  | 27.4  (1.0) | 28.9  (1.4) |  | 30.6  (1.1) | 32.3  (1.1) |  | 29.1  (1.1) | 29.9  (1.1) |  | 26.6  (1.1) | 28.5  (1.2) |
|  | **Spleen** | 27.2  (1.0) |  | 31.6  (1.2) | 33.7  (2.4) |  | 27.4  (1.5) | 29.5  (1.5) |  | 32.4  (1.3) | 34.7  (0.8) |  | 29.2  (1.1) | 31.7  (0.5) |  | 26.4  (1.7) | 29.1  (1.0) |
| ***cd86*** | **PB** | 27.0  (0.5) | 29.8  (0.5) | 30.2  (1.1) | 31.4  (1.3) | 29.6  (0.7) | 29.0  (1.1) | 30.2  (1.1) | 30.0  (0.3) | 30.3  (1.6) | 33.2  (1.5) | 30.3  (0.3) | 30.5  (1.7) | 33.5  (1.6) | 29.8  (0.5) | 29.0  (1.3) | 30.6  (0.8) |
|  | **HK** | 27.8  (0.7) |  | 29.9  (0.8) | 31.6  (1.6) |  | 29.0  (1.0) | 30.6  (1.0) |  | 30.7  (1.3) | 31.6  (2.2) |  | 30.3  (0.)8 | 32.6  (2.0) |  | 29.4  (1.2) | 30.8  (1.5) |
|  | **Spleen** | 28.2  (0.3) |  | 30.7  (1.2) | 30.8  (1.3) |  | 29.2  (0.9) | 29.2  (0.5) |  | 29.3  (0.9) | 31.8  (1.1) |  | 30.9  (1.4) | 32.0  (0.4) |  | 29.8  (1.4) | 30.1  (0.6) |
| ***cd40*** | **PB** | 22.8  (0.7) | 26.7  (0.4) | 26.3  (1.4) | 28.8  (1.2) | 26.9  (0.1) | 24.8  (0.8) | 25.7  (1.6) | 26.9  (0.6) | 26.6  (1.6) | 30.1  (1.6) | 26.2  (0.1) | 25.5  (1.2) | 26.1  (1.4) | 26.0  (0.4) | 24.0  (0.8) | 24.5  (0.8) |
|  | **HK** | 22.0  (0.8) |  | 25.9  (0.6) | 26.9  (2.0) |  | 23.7  (0.6) | 24.7  (1.0) |  | 26.1  (0.8) | 27.7  (1.0) |  | 24.4  (0.5) | 25.2  (1.3) |  | 23.5  (0.7) | 24.3  (0.7) |
|  | **Spleen** | 23.1  (0.8) |  | 27.1  (1.0) | 29.5  (2.7) |  | 24.5  (1.1) | 26.1  (1.7) |  | 27.5  (1.6) | 30.4  (0.9) |  | 24.6  (0.6) | 26.8  (0.8) |  | 23.7  (0.9) | 25.3  (1.3) |
| ***mhcII*** | **PB** | 20.7  (0.4) | 22.7  (0.3) | 23.8  (0.9) | 25.2  (0.7) | 22.2  (0.4 | 22.9  (0.7) | 22.7  (0.4) | 22.9  (0.6) | 24.1  (1.1) | 26.4  (0.4) | 22.1  (0.4) | 23.3  (1.0) | 23.5  (0.4) | 21.8  (0.3) | 22.4  (0.9) | 22.0  (0.5) |
|  | **HK** | 20.9  (0.8) |  | 23.6  (0.5) | 24.2  (1.7) |  | 22.2  (0.5) | 22.3  (0.8) |  | 22.5  (0.8) | 24.6  (1.5) |  | 22.2  (0.5) | 22.8  (1.4) |  | 23.7  (0.5) | 22.1  (1.3) |
|  | **Spleen** | 20.9  (0.8) |  | 24.4  (0.6) | 25.2  (1.3) |  | 22.9  (0.5) | 22.9  (0.8) |  | 23.8  (0.8) | 26.1  (0.7) |  | 23.0  (0.9) | 23.4  (0.3) |  | 22.8  (0.8) | 22.4  (0.8) |
| ***ifna1*** | **PB** | 32.5  (0.7) | 32.5  (0.4) | 33.9  (1.5) | 36.8  (0.4) | 32.8  (0.5) | 32.0  (1.0) | 33.0  (1.0) | 32.4  (0.5) | 34.3  (1.6) | 37.5  (0.2) | 30.3  (0.2) | 32.7  (2.4) | 33.7  (0.6) | 30.5  (0.3) | 30.5  (1.7) | 31.0  (0.4) |
|  | **HK** | 32.9  (0.7) |  | 33.7  (0.7) | 36.5  (0.9) |  | 31.7  (1.2) | 33.1  (0.9) |  | 33.3  (0.5) | 36.8  (1.3) |  | 31.8  (0.7) | 34.0  (0.6) |  | 29.3  (1.2) | 32.0  (1.1) |
|  | **Spleen** | 33.1  (0.6) |  | 33.5  (0.6) | 37.7  (1.7) |  | 31.2  (2.4) | 34.0  (1.8) |  | 34.4  (1.3) | 38.6  (0.9) |  | 31.4  (1.0) | 36.3  (0.8) |  | 28.4  (2.0) | 32.5  (0.5) |
|  |  | PB | ND | ND | ND | ND | ND | ND | ND | ND | ND | ND | ND | ND | ND | ND | ND |
| ***ifnb*** | **HK** | ND |  | ND | ND |  | 33.6  0.8 | ND |  | ND | ND |  | ND | ND |  | 31.5  (0.7) | 33.8  (1.6) |
|  | **Spleen** | ND |  | ND | ND |  | 34.5  (1.8) | ND |  | ND | ND |  | ND | ND |  | 33.1  1.3 | ND |

**Reference**

1. Pfaffl, M. W. A new mathematical model for relative quantification in real-time RT–PCR. *Nucleic Acids Res.* **29**, 2002-2007 (2001).
